# Cost of care for persons with dementia: using a discrete-time Markov chain approach with administrative and clinical data from the dementia service Centres in Austria

**DOI:** 10.1186/s13561-020-00285-w

**Published:** 2020-09-14

**Authors:** Alexander Braun, Paulina Kurzmann, Margit Höfler, Gottfried Haber, Stefanie Auer

**Affiliations:** 1 Institute for Health Care Management, University of Applied Sciences Krems, Piaristengasse 1, AT-3500 Krems, Austria; 2 Department for Economy and Health, Danube University Krems, Dr.-Karl-Dorrek-Straße 30, AT-3500 Krems, Austria; 3 Department for Neurosciences and Preventive Medicine, Danube University Krems, Dr.-Karl-Dorrek-Straße 30, AT-3500 Krems, Austria; 4 Oesterreichische Nationalbank, Josefplatz 1, AT-1015 Vienna, Austria

**Keywords:** Cost and cost analysis, Dementia, Discrete-time Markov chain, Administrative data, Cost simulation, *H42, I11, I18, J14.*

## Abstract

**Background:**

There is growing evidence that the cost for dementia care will increase rapidly in the coming years. Therefore, the objective of this paper was to determine the economic impact of treating clients with dementia in outpatient Dementia Service Centres (DSCs) and simulate the cost progression with real clinical and cost data.

**Methods:**

To estimate the cost for dementia care, real administrative and clinical data from 1341 clients of the DSCs were used to approximate the total cost of non-pharmaceutical treatment and simulate the cost progression with a discrete-time Markov chain (DTMC) model. The economic simulation model takes severity and progression of dementia into account to display the cost development over a period of up to ten years.

**Results:**

Based on the administrative data, the total cost for treating these 1341 clients of the DSCs came to 67,294,910 EUR in the first year. From these costs, 74% occurred as indirect costs. Within a five-year period, these costs will increase by 7.1-fold (16.2-fold over 10 years). Further, the DTMC shows that the greatest share of the cost increase derives from the sharp increase of people with severe dementia and that the cost of severe dementia prevails the cost in later periods.

**Conclusion:**

The DTMC model has shown that the cost increase of dementia care is mostly driven by the indirect cost and the increase of severity of dementia within any given year. The DTMC reveals also that the cost for mild dementia will decrease steadily over the time period of the simulation, whereas the cost for severe dementia increases sharply after running the simulation for 3 years.

## Introduction

In Austria, 145,431 persons are currently living with dementia [1]. Following the trend of demographic change, the number of affected persons will double every twenty years [2]. The costs of illness for dementia in Austria were estimated in the year 2009 at 2.9bn EUR [3, 4]. There is overwhelming evidence that in ageing societies the cost for dementia care will increase rapidly [4–8]. However, especially in German speaking countries, little information about stage-specific cost of care is currently available. Even less information on the cost of community care settings is available and none of the studies take into account the fact of the progressive nature of dementia [9, 10].

A recent study by Schwarzkopf et al. estimates the cost for dementia care by using administrative data from the German statutory insurance, coming to the result that the average annual cost for care for a person with dementia is approximately 47,561 EUR, of which more than 80% are informal costs from care provided by relatives [11]. Also, Schulenburg et al. showed that the cost of Alzheimer’s dementia are varying significantly between mild and severe dementia [12]. These findings highlight that the stage of dementia is one of the main cost drivers [10, 13]. Overall, it seems to be clear that especially the increase of assistive services due to the severity of dementia is a leading cause of increasing cost [13–16]. Schaller et al. state that the care setting and the cost perspective combined with the progressive nature of dementia are relevant drivers for the cost of care, while Jones et al. showed that the developing functional limitations due to dementia result in the societal cost increase per dementia patient of more than 800 EUR per month [13, 15]. Scuvee-Moreau et al. argue that the cost of dementia care does not increase in a linear manner from stages of mild to severe dementia but they describe a parabolic relationship [17]. This means that the cost of treating persons with mild and severe dementia are higher than for persons in the moderate stage of dementia. In order to optimally plan the provision of stage-appropriate services, it is crucial to estimate the cost of care over the entire spectrum of dementia severity. Concluding from these findings, we can foresee that healthcare system cost will increase enormously over the coming decades, especially since no cure for dementia is to be expected in the near future. By stressing the three cost drivers—severity level, cost of informal care, and progression of dementia—the cost of ambulant dementia care could be estimated in order to provide a good basis for care provision planning [18]. Furthermore, the progressive nature of dementia has to be taken into consideration to estimate the prospective total cost of care [9, 15, 19–21].

As the methodological literature recommends, economic evaluations of dementia care should take the cognitive function as a crucial part for the cost approximation into account. Also, the variability of cost in different severity levels should considered in economic evaluations. Markov-based instruments are highly indicated because they combine both aspects. Hence, the most studies where retrospective studies, that raise the cost by a cross-section. Markov-based models could prospect the cost by using longitudinal data [9, 20, 22, 23].

In this study, we estimate the time a patient remains or deteriorates in their disease severity level over a discrete period of 1 year. The best approximation to cope with this problem seems to be the model of discrete time series with a discrete-time Markov chain (DTMC) [15, 19, 21, 24–26]. For a health economic analysis, the combination of clinical assessment and administrative cost data from the DSCs brings new insights. Our analysis focuses on the following issues: (1) raising the stage specific cost of care in a sample of home dwelling persons living with dementia, (2) the progression of dementia as observed under the influence of longitudinal treatment with non-pharmacological methods, and (3) using the clinical progression as basis for a Markov-based simulation framework to simulate the cost development of DSC services in Austria.

## Methods

### Study sample selection and database

Data were collected within the model of the DSC in Upper Austria, a federal state in Austria with approximately 1.5 million inhabitants and having Linz as the capital city. A DSC is defined as a multicomponent, low threshold, ‘one-stop shop’ psychosocial support model, addressing the needs of persons with dementia and their family carers and support providers [27]. The main goals of the DSC care model are early detection of dementia, delaying the institutionalization of persons with dementia and reducing the burden of support providers, by providing psychological screening for symptoms of dementia and dementia severity. Persons are assessed at baseline and every year thereafter. For the clinical assessment, the internationally used Global Deterioration Scale (GDS) is utilized [28]. The GDS is a 7-point scale with satisfactory validity (r = .86) and reliability (r = .92). Within the GDS, each stage is numbered (1–7), so that stages 1–2 represent pre-dementia and stages 3–7 full-blown dementia.

### Description of data

Data from the longitudinal DSC database (DEMDATA) were used. The data are stored in a secure open source technology database. Cognitive test data and medical data are entered by clinical psychologists performing the tests. As persons move into a resident home or die, the follow-up from the DSC ends.

**Fig. 1:**
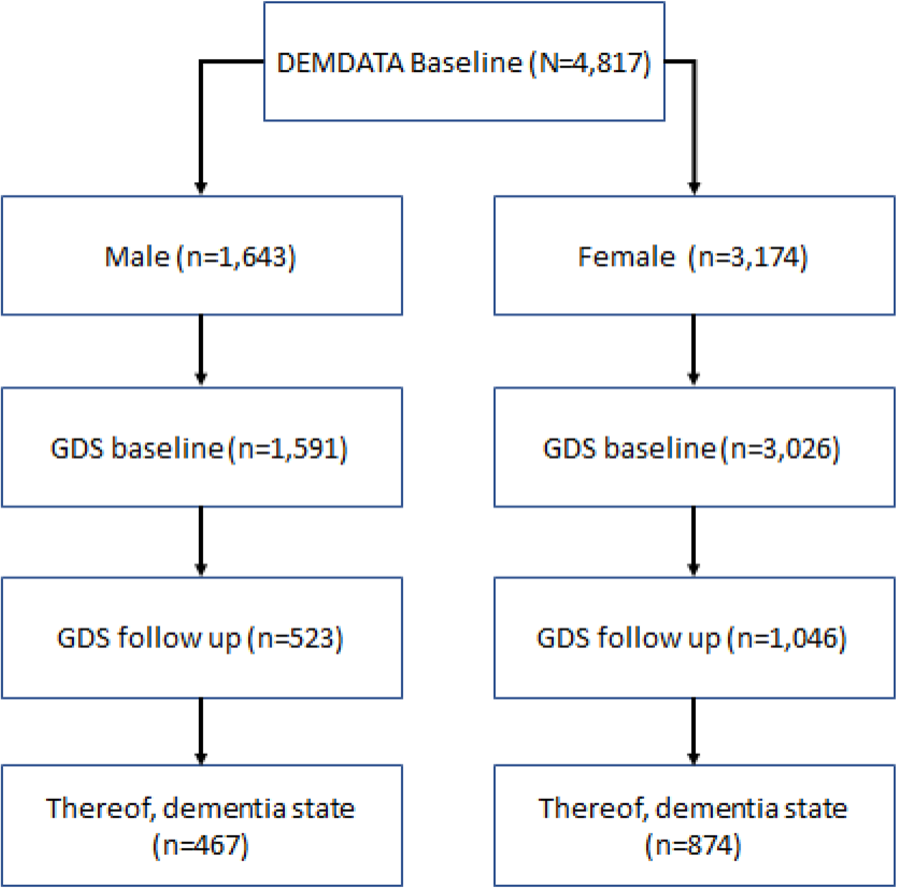
Flow chart of sample selection

In total, baseline data on 4817 clients (3174 females) were available for analysis. At baseline, the average age of the clients was 77.9 years (*SD* = 9.7). Most of the participants (48.9%, *n* = 2354) had completed compulsory school or an apprenticeship (28.3%, *n* = 1361). A further 11.2% (*n* = 539) had no compulsory education, while 5.7% (*n* = 274) had completed some tertiary education. For 6.0% (*n* = 289) of the clients, no information on their educational level was available. The average score of all the clients on the GDS was 4.1 (*SD* = 1.2). At the time of baseline testing, most of the clients (60.9%, *n* = 2931) were not receiving any long-term care benefit, while only 8.5% (*n* = 407) reported to be receiving care benefit. From the remaining 30.7% (*n* = 1479) of clients, no information about care level was available. As not all persons were assessed using the GDS in a follow-up measurement in a year, the sample size for running the simulation is 1341 (Fig. 1). For descriptive statistics and special inferences, we used the statistical program SPSS (IBM, version 25). The statistical simulation was done with the ‘markovchain’-package from the open source statistics program R (Vienna: version: 3.5.1).

### Cost assessment

#### Direct cost

As direct costs of treatment, we identified two main cost sources: (1) the treatment costs that are incurred from the DSC itself, and (2) the costs that are reallocated by statutory long-term care benefit (*Bundespflegegeld*) from the government. Hence, the cost for diseases without an effective pharmacological treatment is mostly deriving from time of (self) care, as methodological costing frame, the patient time cost approach is applied [29, 30]. The direct average treatment costs that were raised by the accounting reporting system from the DSC were 3533.74 EUR per client in 2018, irrespective of the level of dementia severity. In Austria, the long-term care benefit is a non-means-tested service and is based on the hours of care needed—formal, social, and informal—for the patients and should cover parts of the informal costs. Basic to this assessment is the time needed for carers, with the minimum time requirement being strictly defined by a seven-stage scheme. When the caring needs exceed 180 h per month and exceptional tasks are needed, a higher level of the allowance scheme is applied. Due to the ascertained care need, the nursing care allowance distributes their benefits in an upwelling payment scheme. Using this scheme as the costing basis, we had a chance to determine the hours that were needed for taking care of a person with dementia and could infer the direct cost that the Austrian government allocates for people with dementia.

The empirical results show a weak correlation between the stage of GDS and the level of long-term care benefit (Spearman’s rho: .207; *p*-value: < .05), and we are thus using this correlation as basic. Hence, we operationalized the model as follows: clients at GDS stage 3 or 4 received an average of 564.70 EUR per month, clients at GDS 5 got 920.30 EUR, and those at GDS 6–7 got 1487.50 EUR per month (long-term care benefit scheme in the payment year 2018).

#### Indirect cost

Calculating indirect cost is quite a struggle for older patients [31–34]. The indirect costs are defined as those that came up with the care by decreasing productivity (either clients or relatives). As most clients are retired, the indirect costs are mostly derived for informal care costs. Two different methodological approaches are known for raising this kind of indirect cost: the human-capital approach and the friction cost method [35]. As far as Austria is concerned, care for patients with dementia is mostly done by relatives of around the same age, and a strong rational exists for raising the cost of informal care by the human-capital-approach [32, 36]. But as the long-term care benefit covers a part of all costs for long-term care—even the informal care—we avoid double costing in that manner and subtract the direct costs of the long-term care benefit from the higher estimated cost for informal care and take the difference as indirect cost. Nevertheless, the problem of the opportunity cost in dementia care is that older people are not active in the labor market anymore and have therefore no real income loss. The economic methodology favors the opportunity costs method, which arise due to an income loss of the caregiver because of caring tasks and take the loss of free time due to caregiving as its economic base [29, 30, 33]. In this respect, the opportunity costs were calculated as the hours of care multiplied by the average gross market wage.

Another approach would be to take the replacement cost for a professional caregiver into account [6, 32, 34, 37]. The methodology of replacement costs argues moreover that the average labor costs could underestimate the real cost and the valuation of working hours should be undertaken by the price of professional caregiving and would therefore be much higher than the opportunity cost valuation.

Considering these assumptions, we calculated the indirect cost as the labor cost, including the income added-cost where the employer’s taxes, and other costs such as vacation days (25 days), public holidays (11.2 days), average sickness leave (6.3 days), and social security contributions of the employer (22.51% of the gross income) are added onto the gross income. These cost adaptions are based on the income statistics from 2017 [38]. Thus, the official statistics could be used by taking a closer look at the mean gross income, which has to be adapted to the employers on-cost (opportunity-cost-valuation: 23.77 EUR/h) [38]. To compare this cost approximation with the replacement cost, we also calculated the costs for professional caregiving in Upper Austria. As the supply of outpatient dementia care in Upper Austria is based on few providers, it makes sense to calculate the replacement costs by using collective bargaining agreements from the largest suppliers of ambulant dementia care and labor unions in Upper Austria as basic (Caritas, Diakonie, Red Cross, charity and private suppliers). Since there is quite a high penetration of collective agreements for nurses in Austria, the calculation could also be done with the average income of a registered nurse as basic (replacement-costs-valuation: 32.29 EUR/h). Hence, we are using the hours given by the long-term care benefit scheme as the basis for needed hours of care. To cope with the difference of valuing the hourly wages with replacement or opportunity cost, we undertake a one-way-sensitivity-analysis.

Thus, we calculate these indirect costs on the basis of the level of the detected long-term care benefit and multiply the needed hours for care by 140 h per month for mild dementia and 180 h per months for moderate dementia. As the long-term care benefit scheme defines higher levels of care needs specific to extraordinary tasks, we used as the calculation basis 180 h per month where formal care of a registered nurse is obligated for severe dementia and used therefore the replacement-cost-valuation as basic. As the fundament for the simulation model in mild and moderate forms we used the opportunity-cost-valuation. This means that we used the higher hourly labor cost only as basic to estimate the indirect cost for patients with severe dementia.

As can be seen in Table 1, the total cost differs between 33,157.20 EUR per year for mild dementia, 40.299,60 EUR per year for moderate dementia, and 51,896.40 EUR per year for severe dementia. To discount future preferences, we used a discount rate of 5% [39].

**Table 1:** Cost Basics

Type of cost	Mild	Moderate	Severe
**(1). Treatment cost**^a^	3533.74 EUR	3533.74 EUR	3533.74 EUR
**(2). Long-term care benefit (*Bundespflegegeld*)**^b^	6776.40 EUR	11,043.60 EUR	17,850.00 EUR
**(3). Informal Care Costs**^**c**^	39,933.60 EUR	51,343.20 EUR	69,746.40 EUR
**(4). Indirect Costs (3)–(2)**	33,157.20 EUR	40,299.40 EUR	51,896.40 EUR

Sources: own calculations based on ^a^administrative data from the DSC, ^b^the long-term benefit scheme (Bundespflegegeld) ^c^income statistics from the statistic board Austria (Statistik Austria), long-term benefit scheme (Bundespflegegeld) and collective bargaining agreements for professional caregivers)

### Markov chain

To assess the decline of cognitive functions due to dementia, we have taken into consideration that the level of dementia is getting worse over time. Therefore, a DTMC will be coping with that special issue of this disease. In this respect, the DTMC is defined as:

1 $$ \Pr \left({\mathrm{X}}_{\mathrm{n}+1}={\mathrm{x}}_{\mathrm{n}+1}|{\mathrm{X}}_1={\mathrm{x}}_1,{\mathrm{X}}_2={\mathrm{x}}_2,\dots, {\mathrm{X}}_{\mathrm{n}}={\mathrm{x}}_{\mathrm{n}}\right)=\Pr \left({\mathrm{X}}_{\mathrm{n}+1}={\mathrm{x}}_{\mathrm{n}+1}|{\mathrm{X}}_{\mathrm{n}}={\mathrm{x}}_{\mathrm{n}}\right) $$

The Markov process is working with transitions from a property state X_n_ and examines how this state will change in a time period (X_n + 1_), whereas, a set of states S = (s_1i_,s_2_,…, s_3_) are defined by the GDS classification and show how the dementia severity is progressing within a given year. We operationalize this as follows: mild dementia: GDS 3–4, moderate dementia: GDS 5, and severe dementia: GDS 6–7. By using the empirical data from the dataset of the DSC, we calculate the transition probabilities p_ij_ to move from state s_i_ to state s_j_ in one step is named transition probability. This transition probability is calculated within the different severity level:

2 $$ {\mathrm{p}}_{\mathrm{i}\mathrm{j}}=\Pr \left({\mathrm{X}}_1={\mathrm{s}}_{\mathrm{j}}|{\mathrm{X}}_0={\mathrm{s}}_{\mathrm{i}}\right) $$

**Fig. 2:**
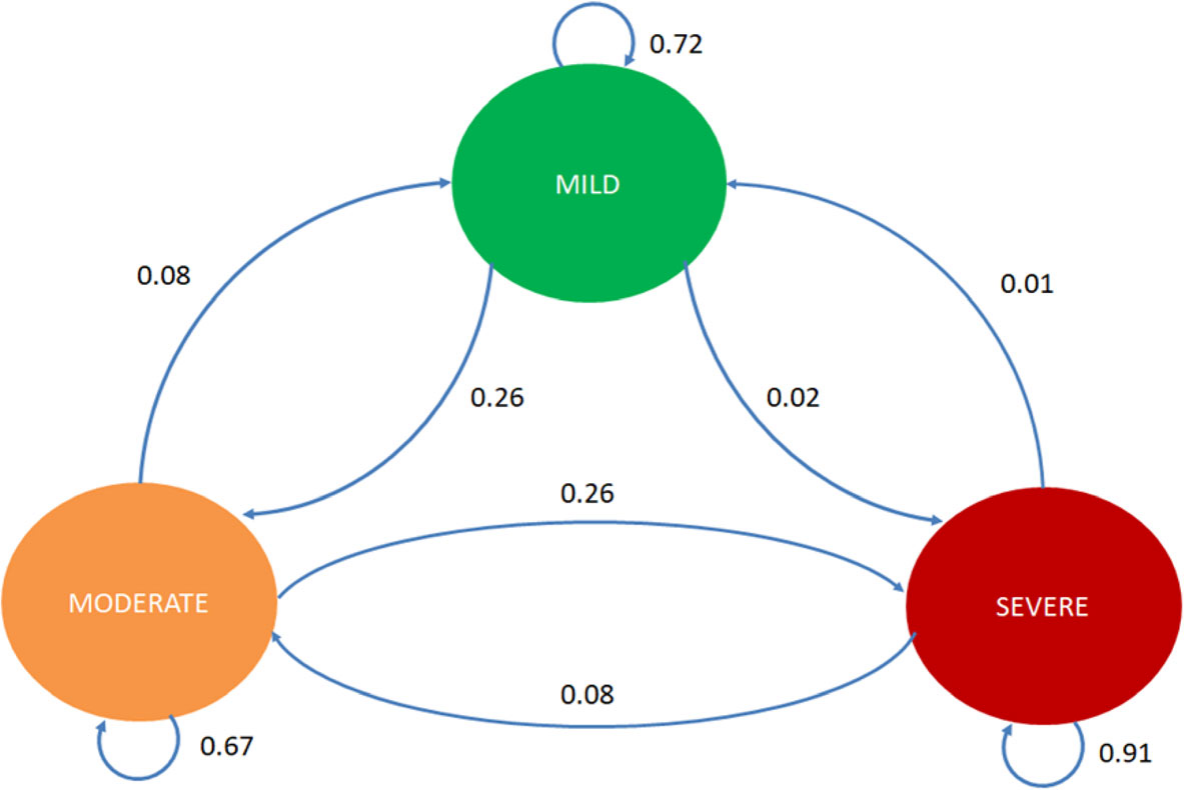
Markov Chain Model

The margin of the transition probabilities was estimated with the maximum likelihood method and was conducted with the assessments of the patients at their first visit and a follow-up measurement after a year. The Markov property states are defined as the distribution of the forthcoming level of dementia (t + 1) in relation to the current state X_n_. Figure 2 shows the following transitions within these three states. Hence, by using microdata, we could estimate the DTMC model with the lower and upper endpoint from the 95%-confidence interval (95%-CI) via maximum-likelihood (Table 2 in Appendix).

**Fig. 3:**
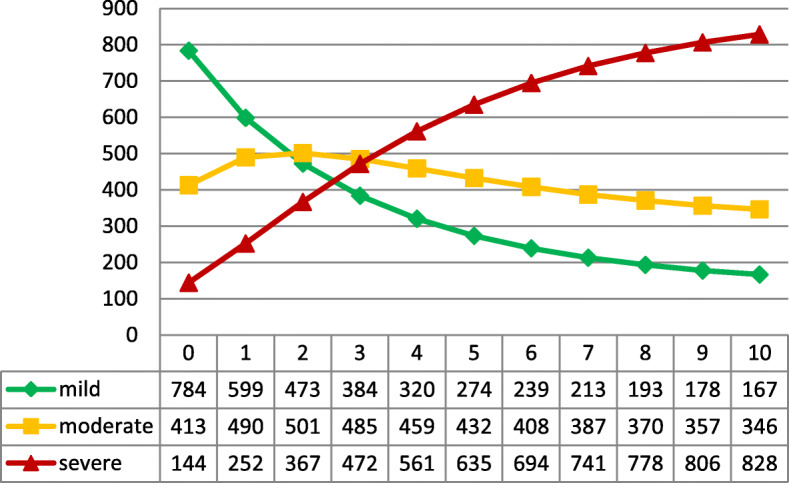
Patient transitions over time in severity levels

For running the DTMC simulation, we used the cohorts of the DSC: 784 clients with mild dementia, 413 clients with moderate dementia, and 144 clients with severe dementia. Overall, we ran 10 iterations, which show how many people stay and move over a period of ten years in each level of severity. Our computation shows that there is no absorbing and steady state within our transition matrix. How the patients progress over this period is shown in Fig. 3.

## Results

**Fig. 4:**
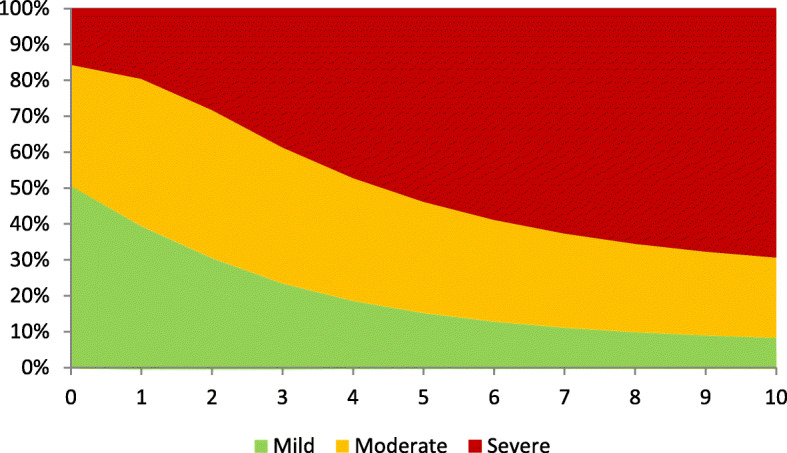
Percentage of cost share over years in GDS level

The costing simulation shows that in the first years the costs are mostly derived from a mild stage of dementia. As we see in Fig. 4, nearly 50% of the total costs per year are costs for treating mild forms of dementia.

**Fig. 5:**
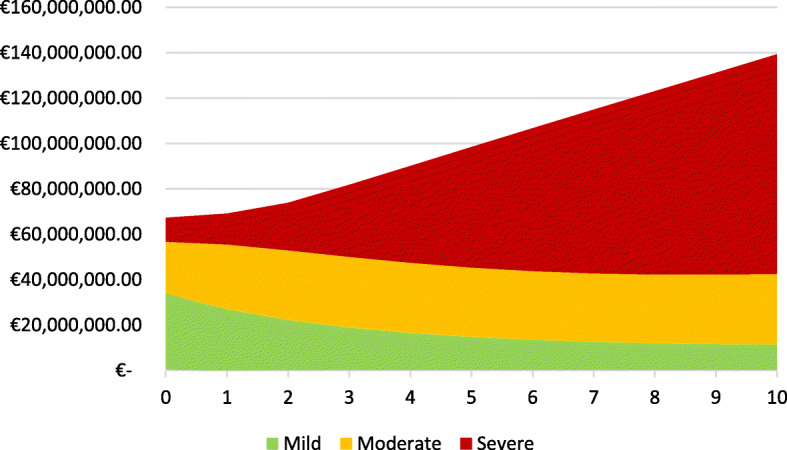
Total cost development in mio. EUR within year

Over time, this proportion is constantly decreasing and the moderate and severe dementia cases are taking over. After 3 years, the cost of severe dementia treatment is increasing sharply and is going to take one-fourth of all costs. Nevertheless, we also see that the costs for the moderate level are stagnating around 30.0 to 38.9% of the total cost. In total, it is clear that the costs are increasing steadily from 67,294,910 EUR per year in period one up to 139,324,193 EUR per year in a ten-year period (Fig. 5). There is evidence that the cost proportion of severe dementia to the total cost increases from 15.7% at the beginning to 35.8% after 5 years and goes up to 52.5% after 10 years, while the proportion of mild dementia declines from 50.6% to 18.0% within the same time period. Another alarming result of our simulation is that the summarized cost for dementia care will increase by 7.1-fold within just 5 years and will climb to a 16.2-fold increase after 10 years.

### Sensitivity analysis

**Fig. 6:**
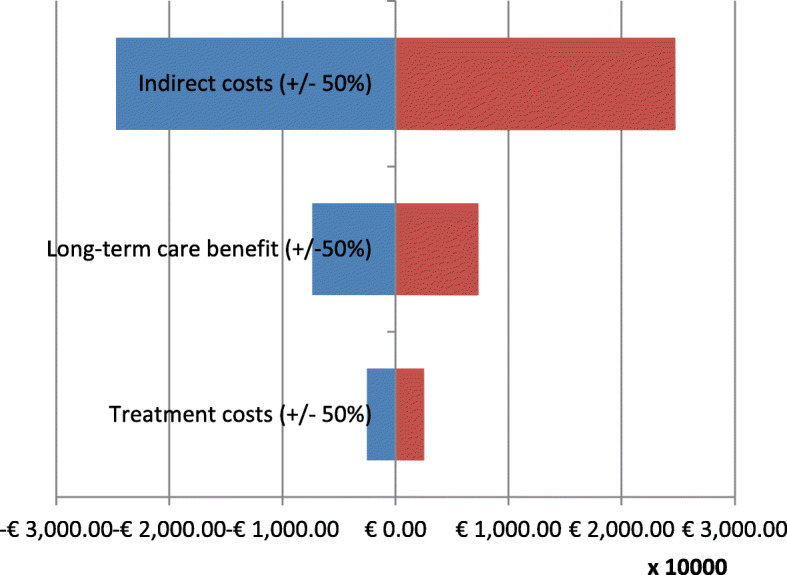
Tornado diagram based on the first year total cost (One-way sensitivity analysis)

After the discussion as to which costing method should be applied for raising the indirect cost for dementia care patients, we undertook a one-way sensitivity-analysis to cope with the uncertainty of the costing issues. As a basis, we vary the direct and indirect cost by ±50%. All variations are shown in a tornado diagram (Fig. 6).

As mentioned before, most of the uncertainty and variance are based on the indirect cost. This could be easily explained by the high share of indirect cost on the total costs. A highlighted result of the sensitivity-analysis is that the variation of the long-term care benefit by ±50% has more effect than the treatment cost of the DSC.

## Discussion

The rationale of this study was to calculate the cost of outpatient dementia care over the severity level. As the research aims also stated, we wanted to estimate the cost progression in parallel with the transition of the severity levels of dementia. We could show that the cost increases rapidly just by the normal progression of dementia and beyond this informal care is the main cost driver within the cost share.

The results of our economic simulation are quite similar to those of other studies [6, 8, 10, 11, 22, 40]. Overall, we came to the same result on the issue of how the disease severity affects the cost of treatment. In agreement with other studies, we also had a proportion of direct cost of around 25%. We also found that the cost of treating severe dementia is nearly twice as high as the cost of treatment for a mild form [6]. It is also evident that the cost variation is mostly influenced by the time that is needed for informal care, and the direct cost tends to be a quite small fragment of the total cost for dementia care [6, 10, 11, 40]. In addition, our study showed that the progression in terms of the severity has a big impact on the cost. Similar to Neumann et al. and Spackman et al., the Markov chain simulation brought good insights into the question of the prospect cost distribution [21, 25]. As we have shown, this increase is mostly driven by the fast worsening of the dementia. Moreover, our findings also suggest that there is a slight movement from mild to moderate dementia and vice versa, but we did not find large possibilities to improve cost performance when the GDS indicates a severe level.

## Conclusion

Just taking the result of the progression of dementia into account, it is quite alarming for policy makers, because the current simulation leads to the assumption that the cost for dementia care will increase quite rapidly once dementia is initially diagnosed. We also want to remind the reader that this cost simulation is limited to outpatient treatment and could not make any assumptions on the issue of resident care patients, nor could we argue whether the care of DSC would save money for the society because their special care could retard the progression of dementia. Hence, further research is planned. With the proportion from informal to direct cost of our study showing 82.8%, our result is quite similar to the results of Schaller et al. [13]. This could be explained by the issue that our study did not involve costs from hospital stay and non-medical care. In this respect, we face quite high differences in direct cost to other studies. Nonetheless, it is obvious that the high share of informal costs will be an economic burden for families and other care persons [13].

### Strengths and limitations

One strength of this study is the large number of participants that were assessed twice using the GDS, so that we could calculate the transition probabilities with relative reliability. But we also have concerns about the generalizability of our results because of two reasons: (1) The DSCs are organized as healthcare centers, which are free of charge for the patients. Due to this aim, we could not infer how strong the external validity of our study sample is. Hence, we did not infer the external costs like hospitalization, emergency visit or drug use. (2) Any randomization or control group was prohibited due to ethical and political issues and merging our data with data from statutory health insurances was not possible. Nonetheless, the treatment in the DSC is focused on the social support of clients and their relatives, a patient time cost approach could overcome these limitations by using patient time costs as a basis. Nonetheless, we can confidently stress that the description of our study sample has a high similarity with other comparable study settings, which suggests a fair external validity [11, 12, 21, 23, 25]. From the costing side, we had to deal with a high variation, especially for the labor cost, and as Schaller et al. and Deb et al. stated, there is yet no homogeneous methodology established [13, 41]. We tried to overcome this issue by moderating the cost valuation in the model and using a sensitivity analysis to cope with this uncertainty. In this respect our study reveals also the problem of deciding whether gross or net-cost should be taken into consideration. Hence, our perspective was focused on the cost from a society perspective, so we decided to take direct associated gross cost with the DSC into consideration. In Austria, as well as in other federally oriented care systems, we are facing a fragmentation of dementia care over the federal policy system and, therefore, a solid database is missing. Nevertheless, subsequent research should try to consider this issue. We also stress that a combination of this prevalence-based model with an incidence-based cost of illness approach could bring up a dynamic cost approximation and could infer the costs to a broad extent, and allows statements on a population-level [41].

Nonetheless, it was not possible to identify the immediate link from direct cost to the DSC needs, except from the cost of the long-term care benefit per patient. This leads to a fuzzy cost approximation, which is concrete on the issue of non-medical cost. Another limitation that occurs due to missing information is about the death of the patients. We suffer from a lack of knowing how long the patients live after the first examination or even if the patients drop out when they go into residency in long-term care. This restricts the explanatory power of the long-term effects because some studies have shown that the mortality of patients with dementia is quite high and after 8.5 years more than 50% of the patients have died [21]. But it is also possible, that we are underestimating the results, especially due to the assumption that in late time periods the end-of-life costs are increasing rapidly [41]. In this respect, we must state that missing information about the reason for dropouts could have biased our cost simulation [26]. Whether this leads to be an over- or underestimation depends on the survival rate, the hospitalization rate and the end-of-life cost and must be answered in following studies. After all, we could also not show whether the DSC decreases the transition probabilities and will for this reason be cost-effective to the standard care. Further research projects are planned to close this gap. This will be part of a further research project and yield new insights into the health economic evaluation of the DSC.

## Data Availability

The datasets used and analyzed during the current study are available from the corresponding author upon reasonable request.

## Appendix

**Table 2:** Transition probabilities for the DTMC model

	Mild (t + 1)	Moderate (t + 1)	Severe (t + 1)
Mild	72.2% [67.2–77.2%]	26.0% [23.0–29.0%]	1.8% [1.0–3.0%]
Moderate	7.5% [5.3–9.7%]	66.6% [60.0–73.2%]	25.9% [21.8–30.0%]
Severe	1.4% [0.0–3.0%]	7.7% [3.9–11.4%]	90.9% [77.9–100.0%]

values in parentheses are the lower and upper endpoint of the estimated 95%-CI

## Abbreviations

DEMDATA: Longitudinal dataset from the Dementia Service Centres
DSC: Dementia Service Centres
DTMC: Discrete-time Markov chain
EUR: Euro
EUR/h: Euro per hour
GDS: Global Deterioration Scale
95%-CI: 95% confidence interval
